# Tobacco smoking, smoking cessation and life expectancy among people with HIV on antiretroviral therapy in South Africa: a simulation modelling study

**DOI:** 10.1002/jia2.26315

**Published:** 2024-06-25

**Authors:** Acadia M. Thielking, Kieran P. Fitzmaurice, Ronel Sewpaul, Stavroula A. Chrysanthopoulou, Lotanna Dike, Douglas E. Levy, Nancy A. Rigotti, Mark J. Siedner, Robin Wood, A. David Paltiel, Kenneth A. Freedberg, Emily P. Hyle, Krishna P. Reddy

**Affiliations:** ^1^ Medical Practice Evaluation Center Massachusetts General Hospital Boston Massachusetts USA; ^2^ Human and Social Capabilities, Human Sciences Research Council Cape Town South Africa; ^3^ Department of Biostatistics Brown University School of Public Health Providence Rhode Island USA; ^4^ Harvard Medical School Boston Massachusetts USA; ^5^ Tobacco Research and Treatment Center Massachusetts General Hospital Boston Massachusetts USA; ^6^ Mongan Institute Health Policy Research Center Massachusetts General Hospital Boston Massachusetts USA; ^7^ Division of General Internal Medicine Massachusetts General Hospital Boston Massachusetts USA; ^8^ Division of Infectious Diseases Massachusetts General Hospital Boston Massachusetts USA; ^9^ Africa Health Research Institute Somkhele South Africa; ^10^ Desmond Tutu Health Foundation, Mowbray Cape Town South Africa; ^11^ Department of Medicine University of Cape Town Cape Town South Africa; ^12^ Public Health Modeling Unit Yale School of Public Health New Haven Connecticut USA; ^13^ Department of Health Policy and Management Harvard T. H. Chan School of Public Health Boston Massachusetts USA; ^14^ Division of Pulmonary and Critical Care Medicine Massachusetts General Hospital Boston Massachusetts USA

**Keywords:** HIV, antiretroviral therapy, tobacco, smoking, smoking cessation, South Africa

## Abstract

**Introduction:**

As access to effective antiretroviral therapy (ART) has improved globally, tobacco‐related illnesses, including cardiovascular disease, cancer and chronic respiratory conditions, account for a growing proportion of deaths among people with HIV (PWH). We estimated the impact of tobacco smoking and smoking cessation on life expectancy among PWH in South Africa.

**Methods:**

In a microsimulation model, we simulated 18 cohorts of PWH with virologic suppression, each homogenous by sex, initial age (35y/45y/55y) and smoking status (current/former/never). Input parameters were from data sources published between 2008 and 2022. We used South African data to estimate age‐stratified mortality hazard ratios: 1.2−2.3 (females)/1.1−1.9 (males) for people with current versus never smoking status; and 1.0−1.3 (females)/1.0−1.5 (males) for people with former versus never smoking status, depending on age at cessation. We assumed smoking status remains unchanged during the simulation; people who formerly smoked quit at model start. Simulated PWH face a monthly probability of disengagement from care and virologic non‐suppression. In sensitivity analysis, we varied smoking‐associated and HIV‐associated mortality risks. Additionally, we estimated the total life‐years gained if a proportion of all virologically suppressed PWH stopped smoking.

**Results:**

Forty‐five‐year‐old females/males with HIV with virologic suppression who smoke lose 5.3/3.7 life‐years compared to PWH who never smoke. Smoking cessation at age 45y adds 3.4/2.4 life‐years. Simulated PWH who continue smoking lose more life‐years from smoking than from HIV (females, 5.3 vs. 3.0 life‐years; males, 3.7 vs. 2.6 life‐years). The impact of smoking and smoking cessation increase as smoking‐associated mortality risks increase and HIV‐associated mortality risks, including disengagement from care, decrease. Model results are most sensitive to the smoking‐associated mortality hazard ratio; varying this parameter results in 1.0−5.1 life‐years gained from cessation at age 45y. If 10−25% of virologically suppressed PWH aged 30−59y in South Africa stopped smoking now, 190,000−460,000 life‐years would be gained.

**Conclusions:**

Among virologically suppressed PWH in South Africa, tobacco smoking decreases life expectancy more than HIV. Integrating tobacco cessation interventions into HIV care, as endorsed by the World Health Organization, could substantially improve life expectancy.

## 1 INTRODUCTION

South Africa is home to over seven million people with HIV (PWH), more than any other country [1]. Improved access to antiretroviral therapy (ART) has increased life expectancy among PWH in South Africa: as of 2010−2014, females and males on ART aged 35y could expect to live an additional 26.1 and 21.1y, an improvement of more than a decade compared to the 2001−2006 period [2]. However, non‐AIDS‐defining illnesses such as cardiovascular disease, cancer and chronic respiratory conditions account for a rising proportion of deaths among PWH on ART [3]. Tobacco smoking, an established risk factor for these diseases, is highly prevalent in South Africa (8% in females and 37% in males in 2016), and approximately 17% of deaths among adults over age 35y are smoking‐attributable [4, 5]. Smoking prevalence is lower in South African females than males due partly to social taboos, although prevalence among females may increase at lower socio‐economic statuses [6, 7]. Among Black South Africans, smoking prevalence is higher among people with middle socio‐economic status and may be higher among some subgroups of PWH compared with the general population [4, 6, 8].

Little is known about the impact of smoking on health outcomes for PWH in sub‐Saharan Africa [9]. Determining the potential life‐year gains from smoking cessation—both at the individual and population levels—could help policymakers decide whether to incorporate smoking cessation interventions into HIV care programmes.

## 2 METHODS

### 2.1 Analytic overview

We used the Cost‐Effectiveness of Preventing AIDS Complications‐International (CEPAC‐I) model, a validated Monte Carlo microsimulation of HIV natural history and treatment [10, 11, 12], to estimate the impact of tobacco smoking and smoking cessation on health outcomes among PWH in South Africa. We focused on people with virologic suppression at model start, given the higher near‐term morbidity and mortality risks associated with unsuppressed HIV compared to smoking [13, 14, 15]. This also reflects the emphasis on attaining virologic suppression in HIV care guidelines [16, 17]. We accounted for subsequent viraemia due to resistance, disengagement from HIV care or incomplete ART adherence. We simulated 18 cohorts (two sexes * three initial ages * three smoking statuses), each homogenous by sex, initial age (35y/45y/55y) and smoking status (current/former/never). We simulated one million people in each cohort to attain stable estimates. We conducted a sensitivity analysis around key smoking‐related and HIV‐related parameters.

There were three main outcomes and comparisons of interest. First, we projected the impact of smoking and smoking cessation on life expectancy (LE, reported as age at death for ease of interpretation by providers and patients, and different from traditional demographic methods which report LE as life‐years from the current age). To accomplish this, we assumed no changes in smoking status during the simulation—that is people who currently smoke (CS) at model start continue to smoke until death, people who formerly smoked (FS) quit smoking upon model start and remain abstinent, and people who never smoked (NS) remain abstinent. We defined life‐years lost from smoking as the LE difference between NS and CS and defined life‐years gained by smoking cessation as the LE difference between FS and CS. Second, we used the model to compare the LE losses from smoking with the LE losses from treated HIV. Third, we estimated the population‐level impact of smoking cessation in terms of life‐years gained if a proportion of virologically suppressed PWH in South Africa stopped smoking.

### 2.2 Model structure

#### 2.2.1 Overview

The CEPAC model simulates PWH on a monthly cycle through stages of disease progression and treatment until death [10, 11, 12]. In this analysis, PWH enter the model with virologic suppression on tenofovir/lamivudine/dolutegravir [16]. At model start, each simulated individual is assigned an initial CD4 count and a probability of continued ART adherence, each based on distributions derived from primarily South African data (Table 1) [18, 19, 20, 21]. Simulated PWH face monthly probabilities of virologic rebound, followed by opportunities to resuppress, or progress to different ART regimens (Table 1 and Table S1). PWH also experience monthly probabilities of disengagement from HIV care (resulting in ART discontinuation) with subsequent opportunities to return to care. CD4 counts influence the monthly probabilities of HIV‐associated morbidity and mortality. There is a monthly sex‐ and age‐dependent probability of dying from causes not typically attributable to HIV/AIDS (e.g. cardiovascular disease, non‐AIDS‐defining cancer, trauma). Model specifications are documented in previous CEPAC analyses [10, 11, 12]. Model details including flowcharts, manuals and sample code are available at https://mpec.massgeneral.org/cepac‐model.

**Table 1:** Selected model input parameters for simulated people with HIV in South Africa

Parameter	Base case value	ART‐naïve scenario analysis	Sensitivity analysis ranges	Source
Mean (SD) initial CD4 count, cells/µl				[18, 19]
Females	789 (318)	432 (206)	100−800	
Males	600 (303)	314 (160)	100−800	
Mean adherence to ART,^a^ %	95.3	90.8		[20, 21, 22]
Proportion with adherence 95−100%	77.1	51.3		
Proportion with adherence 80−94%	17.2	35.8		
Proportion with adherence 66−79%	3.2	7.9		
Proportion with adherence 0−65%	2.4	5.1		
Mean proportion of patients who attain initial virologic suppression on first‐line ART (TDF + 3TC + DTG), %	100	86.6		[20, 23–29]
Adherence ≥ 95%	100	96.4		[20, 23–29]
Adherence 66−94%	100	85.1		[20, 25–27, 30]
Adherence ≤ 65%	100	0		Assumption
Mean monthly probability of subsequent viraemia on first‐line ART, %	0.2	–		
Adherence ≥ 95%	0.2	–		[20, 23, 24, 26–28]
Adherence 31−94%	0.3	–		[31]
Adherence ≤ 30%	18.0	–		[22]
Monthly probability of disengagement from HIV care,^b^ %				[32]
During months 1−12	0.7	1.6	0.0−2.0	
During months 13+	0.7	0.7	0.0−2.0	
Monthly probability of returning to care and resuming ART among people who are lost to follow‐up, %	1.3			[33]
Non‐AIDS mortality hazard ratio, current versus never smoking status, range by age^c^				[14, 15, 34]
Females	1.2−2.3		1.2−3.0	
Males	1.1−1.9		1.2−3.0	
Non‐AIDS mortality hazard ratio, former versus never smoking status, range by age^d^				[15, 34]
Females	1−1.5			
Males	1−1.3			

Abbreviations: ABC, abacavir; ART, antiretroviral therapy; AZT, zidovudine; DRV/r, darunavir/ritonavir; DTG, dolutegravir; IQR, interquartile range; LPV/r, lopinavir/ritonavir; SD, standard deviation; TDF, tenofovir disoproxil fumarate; 3TC, lamivudine.

^a^ Base case adherence distributions were derived from the ADVANCE and NAMSAL randomized controlled trials [20, 21]. ART‐naïve adherence distributions were derived from pharmacy refill records at a rural treatment site in South Africa [22].

^b^ The ART‐naïve cohort has a higher probability of disengagement during the first 12 months after initiating ART, based on a cohort of PWH initiating ART in South Africa [32].

^c^ Base case mortality hazard ratios for people with current versus never smoking status vary by age and were derived from the attributable fraction of deaths due to smoking in South Africa reported by the Global Burden of Disease Study (Supplementary Methods and Table S2) [34]. Sensitivity analysis mortality hazard ratios do not vary by age and were derived from Sitas et al. and Jha et al [14, 15].

^d^ Mortality hazard ratios for people with former versus never smoking status vary by age. Derivations are described in the Supplementary Methods and Table S3 [15, 34].

#### 2.2.2 Smoking‐related mortality

We assumed smoking influences the monthly non‐AIDS mortality probability from age 40y, aligning with data indicating that smoking‐associated mortality becomes more apparent after that age [15, 35]. We assumed the monthly non‐AIDS mortality probabilities for FS are identical to those of CS until 5y after smoking cessation, reflecting findings from cohort studies where smoking cessation benefits did not take effect immediately [15, 35, 36].

### 2.3 Input parameters

Previously published or publicly available data, to be applied as model input parameters and/or for validation, were collected between 1 November 2021 and 31 October 2022. These data sources had been published between 2008 and 2022.

#### 2.3.1 Cohort characteristics

The mean sex‐stratified CD4 count at model start is 789 cells/µl (standard deviation [SD] 318/µl) for females and 600 cells/µl (SD 303/µl) for males, as derived from the population‐based Vukuzazi cohort in South Africa (Table 1) [18]. Adherence‐stratified probabilities of virologic suppression and subsequent viraemia were derived from 48‐ and 96‐week follow‐up data from randomized controlled trials (Table 1 and Table S1) [20, 24–29, 37–40]. Derivation methodologies were described previously [12]. The probability of disengagement from HIV care is 0.7%/month [32]. After 6 months, individuals can return to HIV care by a 1.3%/month background probability or after a new severe opportunistic infection [33].

#### 2.3.2 Smoking‐related mortality

We estimated non‐AIDS mortality rates among PWH in South Africa, stratified by age and sex, based on population estimates and cause‐specific death data [41, 42]. To derive non‐AIDS mortality for CS, FS and NS, we solved equations based on three assumptions. First, the overall non‐AIDS mortality rate is a weighted average of CS, FS and NS mortality rates based on age and sex‐stratified smoking prevalence estimates from the South Africa Demographic and Health Survey (SADHS, Table S4) [4]. We used general population smoking prevalence estimates in the base case because the larger sample size allowed for greater granularity when stratifying by age and sex (established drivers of smoking prevalence in South Africa) [43, 44]. Also, smoking prevalence among virologically suppressed PWH may be lower than among viraemic PWH, and thus closer to the general population smoking prevalence [8, 45]. Second, we assumed the difference in mortality rates between the general population and NS is proportional to the fraction of non‐AIDS deaths attributable to smoking as reported by the Global Burden of Disease study (Supplementary Methods and Table S2) [34]. Third, we assumed FS mortality rates equal the difference in mortality rates between CS and NS multiplied by the fraction of excess non‐AIDS mortality risk (i.e. the risk among CS retained by FS;calculated from age and sex‐stratified hazard ratios [HRs] reported by Jha et al.) (Table S3) [15]. The resulting smoking‐stratified non‐AIDS mortality rates yielded age‐stratified mortality HRs of 1.2−2.3 (females) and 1.1−1.9 (males) for CS compared to NS (Table S2). Details are in the Supplementary Methods.

### 2.4 Model validation methods

We internally validated the proportion‐weighted LE of CS, FS and NS with the model‐projected LE of a cohort without smoking stratifications. We externally validated base case and ART‐naïve LE projections with previously reported South African LE estimates (Supplementary Methods) [2, 46].

### 2.5 Life expectancy losses from smoking and from HIV

To quantify LE losses from smoking, we compared LE of CS with HIV and NS with HIV. To quantify LE losses from HIV, we modelled CS without HIV and compared their LE to that of CS with HIV. The cohort of CS without HIV was identical to the cohort of CS with HIV except that the former had no HIV/AIDS‐related mortality risks. Additionally, we compared LE losses attributable to smoking versus HIV among people with high (>95%) ART adherence and engagement in HIV care during the simulation, which increases the probability of sustained virologic suppression and decreases the probability of AIDS‐related death.

### 2.6 Sensitivity and scenario analysis

We examined the robustness of our findings when varying several smoking‐related and HIV‐related assumptions and parameters. Smoking‐related sensitivity analyses included: (1) FS quit smoking 2, 5 or 10y after model start; (2) smoking‐stratified mortality derivations use non‐age‐stratified low or high mortality HRs for CS versus NS (1.2 [not sex‐stratified], reported in South Africa [14], or 3.0 [females]/2.8 [males], reported in the United States [15]) instead of the age‐stratified smoking‐attributable fraction; (3) use of smoking prevalence estimates specific to PWH from SADHS for derivations of smoking‐stratified non‐AIDS mortality rates (Table S4). HIV‐related sensitivity and scenario analyses included: (1) high (>95%) ART adherence and no disengagement from ART care; (2) higher monthly probability of disengagement from ART care (1.6%/month), as males and PWH who smoke may have higher risks of disengagement [32, 47]; (3) lower or higher CD4 count at model start (200−800 cells/µl); (4) cohorts comprising ART‐naïve PWH who initiate ART at model start and FS quit smoking 2y after model start (Table 1). Finally, we performed a two‐way sensitivity analysis in which we simultaneously varied the mortality HR for CS versus NS and the monthly probability of disengagement from HIV care, accounting for uncertainty around sex‐specific smoking‐associated hazards and engagement in care [32, 35].

### 2.7 Population‐level impact

We estimated the cumulative life‐years gained if 10−25% of currently smoking PWH on ART with virologic suppression stopped smoking now and remained abstinent, compared with continued smoking (proportions based on smoking abstinence results in trials of cessation interventions) [48]. From 2017 South Africa data, we estimated there were approximately 5.32 million PWH between ages 30 and 59y, of which 3.46 million were on ART and 3.02 million were virologically suppressed [49, 50]. Based on SADHS smoking prevalence data, we estimated that 620,000 virologically suppressed PWH currently smoke [4]. We applied the projected life‐years gained by smoking cessation, weighted by current age and sex, to the final population size to estimate the population‐level impact.

### 2.8 Ethics statement

All data utilized in the model were previously published or publicly available. There were no participants and consent was not required. This study was approved by the Mass General Brigham Human Research Committee (Protocol 2014P002708).

## 3 RESULTS

### 3.1 Model validation results

In internal validation, when comparing the weighted LE of CS, FS and NS cohorts with CEPAC LE projections without smoking stratifications, LE differs by less than 0.3y (Table S6). In external validation, base case and ART‐naïve CEPAC LE projections lie between LE estimates from two prior South African studies (Supplementary Results) [2, 46].

### 3.2 Base case

**Figure 1:**
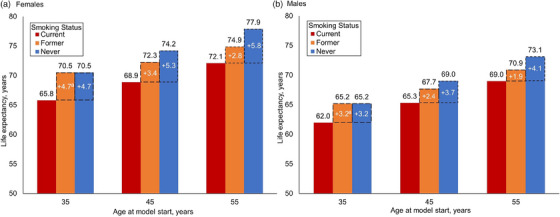
Life expectancy by smoking status among females and males with HIV on antiretroviral therapy in South Africa. Model‐projected life expectancy among (a) females and (b) males with virologic suppression at model start, stratified by age at model start and smoking status. Individuals can experience virologic failure during the simulation. People with current smoking status continue to smoke until death. People with former smoking status quit at model start and remain abstinent. Life expectancy is expressed as the age at the time of death. Numbers in white quantify the difference in life‐years between people with former or never smoking status compared with people with current smoking status. ^a^We assume there is no excess mortality risk among people with HIV who quit smoking before age 40y compared to people with HIV who never smoke [15, 35]. Hence, the life expectancy for people with former smoking status and people with never smoking status is the same among those aged 35y at model start.

**Figure 2:**
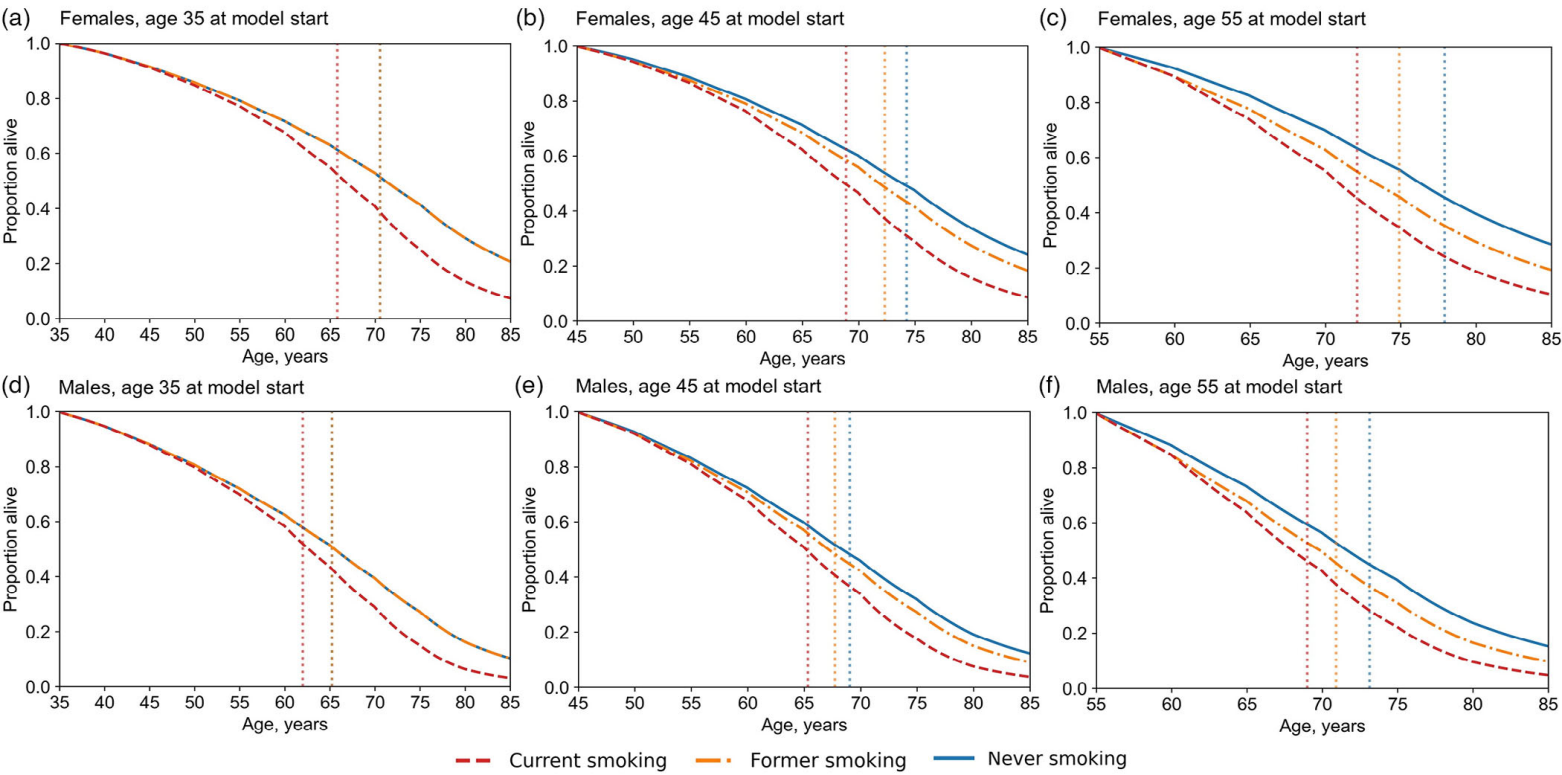
Survival curves stratified by smoking status among females and males with HIV virologically suppressed on antiretroviral therapy in South Africa. Model‐projected Kaplan−Meier survival curves by smoking status (current, former, never) for 35, 45 and 55‐year‐old females and males with virological suppression at model start. Simulated individuals can experience viraemia during the simulation. People who currently smoke continue to smoke until death. People who formerly smoked quit at model start and remain abstinent. Simulations assume that people who quit prior to age 40y experience no excess mortality from smoking [15, 35]. Vertical dotted lines represent the life expectancy for people of each smoking status.

When simulated over their lifetime, LE (reported as age at death) for females with virologic suppression upon model entry at age 45y is 68.9, 72.3 and 74.2y for CS, FS and NS; for males, it is 65.3, 67.7 and 69.0y. CS females and males aged 45y lose 5.3 and 3.7 life‐years from smoking, while females and males who stop smoking at age 45y gain 3.4 and 2.4 life‐years compared to CS. Smoking cessation at younger ages yields greater increases in LE (Figure 1). At older ages at smoking cessation, survival curves of FS shift towards those of CS, and life‐years gained from cessation decrease (Figure 2). Nonetheless, females and males who enter the model at age 55y and stop smoking at that age still gain 2.8 and 1.9 life‐years compared to CS (Figure 1).

### 3.3 Life expectancy losses from smoking and from HIV

As above, simulated 45‐year‐old females and males with HIV lose 5.3 and 3.7 life‐years from smoking (comparing CS with HIV to NS with HIV). Among people without HIV, 45‐year‐old CS females and males have model‐projected LE of 71.9 and 67.9y. Comparing the LE of CS with HIV and CS without HIV, females and males lose 3.0 and 2.6 life‐years from HIV. Among PWH with high ART adherence and engagement in HIV care, simulated 45‐year‐old CS females and males lose 6.5 and 4.5 life‐years from smoking (compared to NS with HIV), whereas they lose 1.6 and 1.4 life‐years from HIV (compared to CS without HIV) (Table 2).

**Table 2:** Model‐projected life expectancy for 45‐year‐old people in South Africa based on smoking status, comparing life‐years lost due to smoking, HIV, and incomplete antiretroviral therapy adherence and engagement in care

	People with HIV, average ART adherence and engagement in care (base case)^a^ A	People with HIV, high ART adherence (>95%) and engagement in care^b^ B	People without HIV C	Life‐years lost from HIV, with high ART adherence and engagement in care^c^ C‐B	Additional life‐years lost from incomplete ART adherence and engagement in care^d^ B‐A	Combined life‐years lost from HIV and incomplete ART adherence and engagement in care^e^ C‐A
**Females**						
Life expectancy, years						
*Current smoking*	68.9	70.5	71.9	1.4	1.6	3.0
*Never smoking*	74.2	77.0	79.1	2.1	2.8	4.9
Life‐years lost from smoking (current vs. never)	5.3	6.5	7.2			
**Males**						
Life expectancy, years						
*Current smoking*	65.3	66.7	67.9	1.2	1.4	2.6
*Never smoking*	69.0	71.2	72.9	1.7	2.2	3.9
Life‐years lost from smoking (current vs. never)	3.7	4.5	5.0			

Abbreviation: ART, antiretroviral therapy.

^a^ People with HIV enter the model at age 45 years and are virologically suppressed, although there is a probability of subsequent viraemia. Mean adherence is 95.3%, and there is a 0.7% monthly probability of disengagement from care [20, 21, 32].

^b^ In these simulations, there is high adherence (>95%) to antiretroviral therapy and engagement in care (0% probability of disengagement from care).

^c^ This is the difference in life expectancy between people without HIV and PWH with high adherence and engagement in care.

^d^ This is the difference in life expectancy between PWH with high adherence and engagement in care and PWH with average adherence and engagement in care.

^e^ This is the difference in life expectancy between people without HIV and PWH with average adherence and engagement in care.

### 3.4 Sensitivity and scenario analysis

We varied input parameters in sensitivity analyses for females and males with HIV aged 35, 45 and 55y at model start. Here, we report results only for 45‐year‐olds; trends remained similar in the other age groups (Table S5).

**Figure 3:**
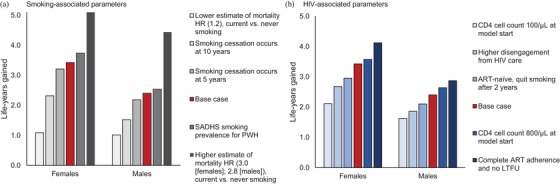
Sensitivity analysis results when varying key parameters: life‐years gained from smoking cessation among 45‐year‐old females and males with HIV on antiretroviral therapy in South Africa. The bars represent the life‐years gained from smoking cessation (the difference in life expectancy between CS and FS) among females and males aged 45y at model start. Results are reported for sensitivity analysis varying assumptions and parameters associated with smoking (Panel A) and HIV (Panel B). In the base case, represented by the red bar in both panels, smoking cessation occurs at model start (age 45y) and all people are virologically suppressed at model start. Abbreviations: ART, antiretroviral therapy; HR, hazard ratio; PWH, people with HIV; SADHS, South Africa Demographic and Health Survey.

When varying smoking‐related parameters, life‐years gained by FS are inversely associated with the time delay to smoking cessation after model start and are sensitive to estimates of smoking‐related mortality and smoking prevalence (Figure 3A and Table S5). For example, females and males aged 45y who stop smoking at model start gain 3.4 and 2.4 life‐years compared to CS, whereas stopping smoking 10 years after model start produces gains of 2.3 and 1.5 life‐years compared to CS.

When varying HIV‐related parameters, reducing competing risks from HIV produces greater LE losses from smoking and greater LE gains from cessation (Figure 3B and Table S5). For example, when all individuals have high ART adherence and engagement in HIV care throughout the simulation, LE for 45‐year‐old females is 70.5, 74.7 and 77.0y for CS, FS and NS; for males, it is 66.7, 69.6 and 71.2y. Life‐years gained from smoking cessation increase compared with the base case (4.1 vs. 3.4 life‐years for females, 2.9 vs. 2.4 life‐years for males).

**Figure 4:**
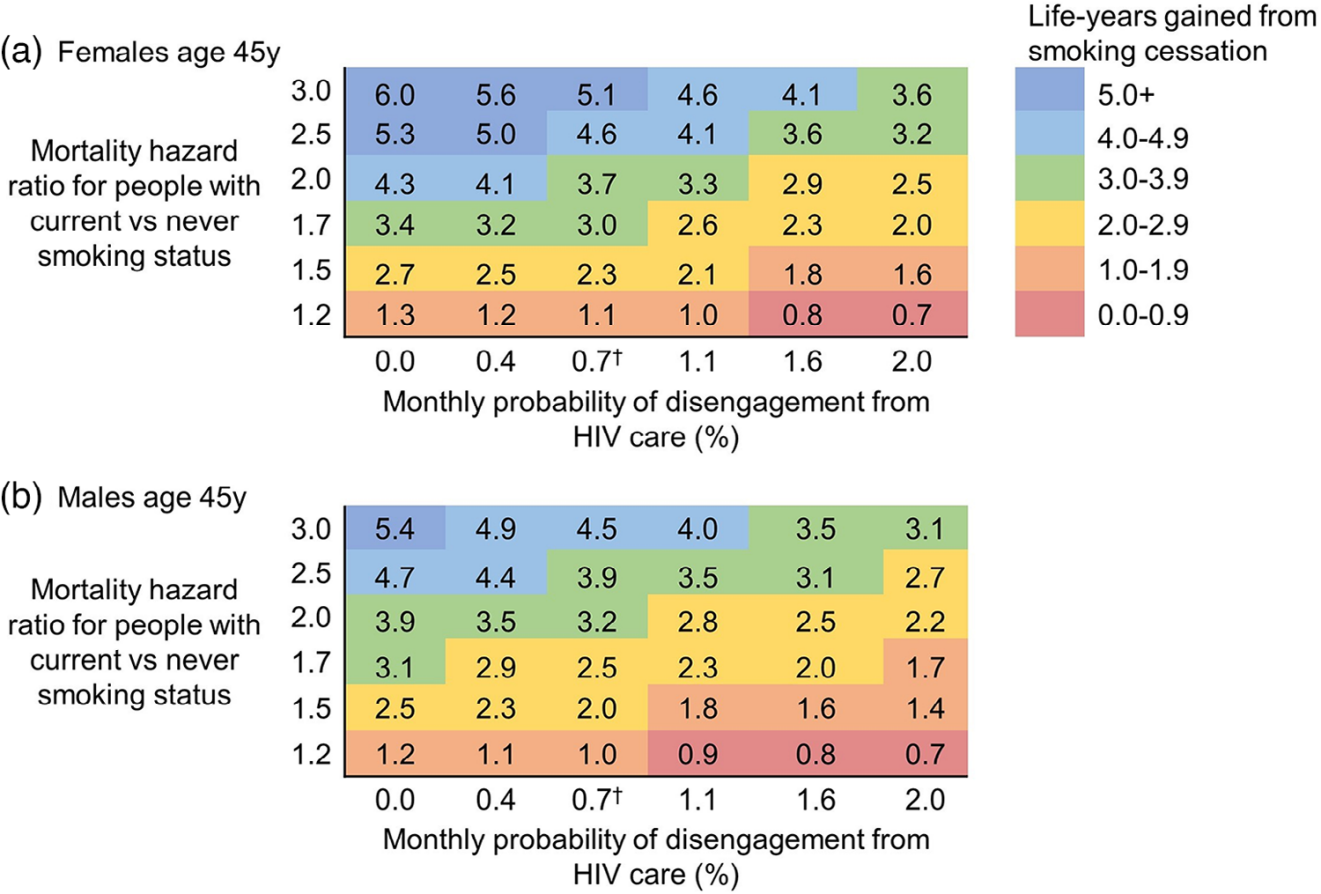
Two‐way sensitivity analysis results: the impact of varying the smoking‐associated mortality hazard ratios and the probability of disengagement from HIV care on life‐years gained from smoking cessation. This figure illustrates the life‐years gained from smoking cessation when varying the mortality hazard ratios for people with current versus never smoking status and the probability of disengagement from HIV care. In this sensitivity analysis, each mortality hazard ratio is applied in a consistent manner to all age groups >40y (i.e. the hazard ratios are not age‐stratified). The “average” hazard ratio in the base case lies between 1.7−2.0 for females and 1.5−1.7 for males, with a 0.7% monthly probability of disengagement from HIV care. ^†^The base case monthly probability of disengagement from HIV care is 0.7%.

In the two‐way sensitivity analysis, as smoking‐related mortality risks increase and disengagement from HIV care decreases (thereby reducing HIV‐related mortality risks), life‐years gained from smoking cessation increase (Figure 4). For example, if the mortality HR for CS compared with NS is 2.5 (closer to that reported in the United States) [15], then life‐years gained by 45‐year‐olds from smoking cessation at that age are 3.2−5.3y for females and 2.7−4.7y for males depending on probabilities of disengagement from HIV care.

### 3.5 Population‐level impact

If 10−25% of 30‐ to 59‐year‐old PWH with initial virologic suppression who smoke in South Africa stopped smoking now and remained abstinent, 190,000−460,000 life‐years would be gained compared to a scenario in which they continued smoking until death.

## 4 DISCUSSION

We projected that PWH in South Africa with virologic suppression who smoke tobacco lose more life‐years from smoking than from HIV. Smoking decreases the life expectancy of PWH by 3−6y, of which 2−5y could be recouped by smoking cessation. Effective smoking cessation programmes could have a profound impact on the health of PWH in South Africa—reducing total life‐years lost by approximately 190,000−460,000 over the lifetime of PWH currently aged 30−59y. Moreover, as access to effective ART increases and HIV‐related mortality decreases, the negative impact of smoking and the benefits of smoking cessation on life expectancy will both increase. These results support the rapid integration of smoking cessation interventions into HIV care in the region.

Our results are robust to assumptions about uncertain smoking and HIV‐related parameters, with at least moderate benefits in life expectancy for 45‐year‐old PWH who stop smoking (≥0.7 life‐years gained) in all scenarios. Model results are most sensitive to smoking‐associated mortality HRs and assumptions about ART adherence and engagement in HIV care. Lower estimates of smoking‐associated mortality HRs in South Africa (which we applied in a sensitivity analysis) may be biased by underreporting of smoking or by uncertain causes of death [14]. Assuming higher hazards of smoking, as reported in the United States, would show more life‐years lost from smoking [15, 35]. The base case smoking‐associated mortality risk, derived from the Global Burden of Disease study, falls between those estimates [34]. Regarding HIV parameters, populations with the lowest risks of HIV/AIDS mortality, including PWH with higher ART adherence, engagement in care and initial CD4 counts, lose the most life expectancy from smoking and gain the most from smoking cessation. ART adherence may be lower among people who smoke and are initiating ART in South Africa (different from our base case population of virologically suppressed PWH) [51]. However, even with less engagement in HIV care and lower initial CD4 count, there remain substantial life expectancy gains from smoking cessation. Though life expectancy gains are higher for people who quit smoking at younger ages, even quitting at age 55y produces gains of >2 life‐years, indicating that smoking cessation interventions should be provided to people of all ages.

These results are consistent with cohort and modelling studies in high‐income countries, which also found more life‐years lost from smoking than from HIV [36, 52]. Compared with findings among PWH in high‐income countries, we found the impact of smoking and smoking cessation to be lower but still substantial in this South Africa‐focused analysis. The discrepancy in individual life‐years lost or gained may stem from the difference in smoking intensity between people who smoke in South Africa, where the majority smoke 1−9 cigarettes/day, and people who smoke in high‐income countries; in the United States, for example, the majority smoke greater than 10 cigarettes/day [4, 53]. It is well‐described that mortality risks increase as smoking intensity increases [35]. In our base case analysis, we applied a lower smoking‐associated mortality HR compared with that applied in US analyses [36, 54]. People in South Africa also experience higher competing mortality risks, such as from non‐AIDS‐related communicable diseases and non‐natural deaths, thereby relatively lessening the deleterious impact of smoking. Nonetheless, population‐level life expectancy gains from smoking cessation compare favourably with gains from other commonly employed interventions in South Africa, such as injectable HIV pre‐exposure prophylaxis and HIV self‐test distribution [55, 56].

The United Nations and the World Health Organization have recommended integrating smoking cessation interventions within HIV care programmes [57]. The relatively high frequency of healthcare system interaction among PWH on ART offers opportunities to deliver smoking cessation interventions to increase life expectancy while solidifying the health benefits achieved by ART. Studies of PWH who smoke in South Africa report that over 80% are interested in stopping smoking, but most are unaware of whom to approach for cessation guidance [8, 58]. Males in South Africa report a lower likelihood than females of being advised to quit smoking [44]. Current South African HIV care guidelines provide no guidance on smoking cessation, and available cessation resources such as the national quit‐line and pharmacotherapy are underutilized [59, 60].

As HIV care infrastructure and ART coverage improve in South Africa and other low‐ and middle‐income countries, these programmes can be leveraged for other interventions to improve the health and survival of PWH. Smoking cessation could be combined with efforts to screen for and treat conditions such as hypertension and diabetes mellitus. Multiple studies across sub‐Saharan Africa have identified feasible methods for non‐communicable disease and HIV care integration, with carefully planned implementation to ensure HIV care quality maintenance, cost‐effectiveness and scalability [61, 62]. While data on smoking cessation intervention efficacy among PWH in sub‐Saharan Africa remain limited, both behavioural and pharmacological interventions have been efficacious in populations of PWH in other settings, and additional studies of smoking cessation interventions for PWH in sub‐Saharan Africa are ongoing [63, 64, 65, 66].

People aged 15−24y have both the highest rates of smoking initiation and HIV incidence in South Africa [49, 67]. Integrating tobacco counselling with HIV prevention and care could reduce smoking initiation. While our study focuses on the benefits of cessation among PWH aged ≥30y in care, future studies could quantify the public health benefits of preventing smoking initiation, especially among youth and young adults.

As with all model‐based analyses, these results should be interpreted in the context of assumptions and data available to populate the model. There is a dearth of nationally representative data on smoking among PWH in South Africa and elsewhere in sub‐Saharan Africa. To define smoking prevalence, we used SADHS data that rely on self‐reported smoking status, which is prone to bias. In the base case, we did not use HIV‐specific smoking prevalence estimates due to less precise estimates for PWH compared with the general population [4]. As shown in the sensitivity analysis using HIV‐specific smoking prevalence, our base case inputs may underestimate the potential life‐years gained by smoking cessation among PWH. We also assumed the effect of smoking on non‐AIDS mortality is the same among virologically suppressed PWH and the general population, but HIV and smoking might cause synergistic harm [68]. Furthermore, we did not specifically account for smoking relapse among people who quit because our aim was to demonstrate the life expectancy benefits that could be achieved if abstinence was maintained. In the “status quo” of our population‐level projections, we assumed static smoking behaviours and did not account for subsequent initiation, cessation or relapse, the probabilities of which are not well‐defined in South Africa. Although smoking‐attributable mortality has been demonstrated extensively elsewhere, there is a chance of unmeasured confounding in the smoking‐related mortality HRs we applied.

## 5 CONCLUSIONS

This modelling analysis projects that smoking substantially decreases the life expectancy of PWH with initial virologic suppression in South Africa and prevents PWH from experiencing the full health benefits of ART. Smoking cessation would decrease mortality and provide major gains in life expectancy. The individual and population‐wide gains in life‐years suggest that smoking cessation interventions should become part of routine care for PWH in South Africa and similar settings. Further research on the feasibility, outcomes and cost‐effectiveness of smoking cessation interventions within HIV care would aid policymakers in implementation.

## COMPETING INTERESTS

KPR reports a grant from the American Lung Association to his institution and royalties from UpToDate, Inc., for authorship of an article about electronic cigarettes. NAR reports a grant to her institution and consulting fees from Achieve Life Sciences, membership in a data and safety monitoring board for Achieve Life Sciences, and royalties from UpToDate, Inc., for authorship of articles about tobacco cessation.

## AUTHORS’ CONTRIBUTIONS

KPF, DEL, NAR, MJS, RW, ADP, KAF, EPH and KPR conceptualized and designed the study. AMT, KPF, RS, SAC, EPH and KPR analysed and interpreted the data. KPF, RS and SAC performed the statistical analysis. AMT, KPF, LD and KPR drafted the manuscript. AMT, KPF, RS, SAC, LD, DEL, NAR, MJS, RW, ADP, KAF, EPH and KPR revised the manuscript critically for important intellectual content. MJS, EPH and KPR obtained funding. LD provided administrative and technical support. KPR supervised the study. All authors read and approved the final manuscript.

## FUNDING

This work was supported by the National Institute on Drug Abuse (R01 DA050482) and the National Institute of Allergy and Infectious Diseases (R37 AI058736) of the National Institutes of Health. MJS receives additional funding from the National Heart, Lung, and Blood Institute (K24 HL166024). EPH receives additional funding from the MGH Jerome and Celia Reich Endowed Scholar Award. The funding sources had no role in the study design, data collection, data analysis, data interpretation, the manuscript's writing or the decision to submit the manuscript for publication.

## DISCLAIMER

The content is solely the responsibility of the authors and does not necessarily represent the official views of the funding sources.

## Supporting information

Supplementary Appendix
The Supplementary Appendix includes model validation details; calculations of fraction of excess mortality risk retained by FS; smoking prevalence by age, sex and HIV status; and derivation of non‐AIDS mortality rates for CS, FS and NS.

## Data Availability

The data that support the findings of this study are included in the article or uploaded as supplementary information.
