# Supplementary material for: Tobacco smoking, smoking cessation and life expectancy among people with HIV on antiretroviral therapy in South Africa: a simulation modelling study
Source: J Int AIDS Soc. 2024 Jun 25;27(6):e26315. doi: 10.1002/jia2.26315 (PMC11197963; doi:10.1002/jia2.26315)
Supplement: Supplementary file 1 — Supplementary Appendix The Supplementary Appendix includes model validation details; calculations of fraction of excess mortality risk retained by FS; smoking prevalence by age, sex and HIV status; and derivation of non‐AIDS mortality rates for CS, FS and NS. [file JIA2-27-e26315-s001.docx]

**Supplementary Appendix**

**Tobacco smoking, smoking cessation, and life expectancy among people with HIV on antiretroviral therapy in South Africa: a simulation modelling study**

Acadia M. Thielking, Kieran P. Fitzmaurice, Ronel Sewpaul, Stavroula A. Chrysanthopoulou, Lotanna Dike, Douglas E. Levy, Nancy A. Rigotti, Mark J. Siedner, Robin Wood, A. David Paltiel, Kenneth A. Freedberg, Emily P. Hyle, Krishna P. Reddy

# **Supplementary Methods**

**Nomenclature for smoking-stratified mortality derivations**

| $P_{N}$ | Proportion of people with never smoking status (NS) |
| --- | --- |
| $P_{F}$ | Proportion of people with former smoking status (FS) |
| $P_{C}$ | Proportion of people with current smoking status (CS) |
| $M_{all}$ | Overall non-AIDS mortality rate |
| $M_{N}$ | Non-AIDS mortality rate among NS |
| $M_{F}$ | Non-AIDS mortality rate among FS |
| $M_{C}$ | Non-AIDS mortality rate among CS |
| $f$ | Fraction of excess non-AIDS mortality risk retained by FS after quitting |
| $\bar{f}$ | Mean value of $f$ after averaging over age at quit distribution of FS |
| $\bar{M_{F}}$ | Mean value of $M_{F}$ after averaging over age at quit distribution of FS |
| $q$ | Age at quit (age at smoking cessation) |
| $AF$ | Fraction of non-AIDS deaths attributable to smoking |
| ${HR}_{C}$ | Hazard ratio for non-AIDS mortality among CS versus NS |
| ${HR}_{F}$ | Hazard ratio for non-AIDS mortality among FS versus NS |
| $w$ | Sample weight |
| $i$ | Subscript denoting individual sample |

## **F****raction of excess mortality risk retained by FS**

We assume the non-AIDS mortality rate among people with former smoking status (FS) can be expressed as a convex combination of the rates among people with never smoking status (NS) and people with current smoking status (CS):

$$\begin{aligned} M_{F}=M_{N}+\left( M_{C}-M_{N} \right)f\#\left( 1 \right) \end{aligned}$$

where $f$ represents the fraction of excess non-AIDS mortality risk retained by people with former smoking status (FS) after quitting. By dividing both sides by $M_{N}$, we can rewrite (1) in terms of mortality hazard ratios compared to NS:

$$\begin{aligned} {HR}_{F}=1+\left( {HR}_{C}-1 \right)f\#\left( 2 \right) \end{aligned}$$

Rearranging, it follows that:

$$\begin{aligned} f=\frac{{HR}_{F}-1}{{HR}_{C}-1}\#\left( 3 \right) \end{aligned}$$

Equation (3) provides a method to estimate $f$ as a function of the mortality hazard ratios for CS and FS compared to NS. We used the all-cause mortality hazard ratios reported by Jha et al. [5] in an analysis from the U.S. National Health Interview Survey to estimate the fraction of excess non-AIDS mortality risk retained by FS based on their age at quit (Table S3).

At the individual level, we used the values of $f$ displayed in Table S3 to determine the excess non-AIDS mortality risk retained by FS as a function of age at quit. However, to derive average mortality rates for CS, FS (combination of all ages at smoking cessation), and NS in South Africa based on population-level data (described below), we determined the average value of $f$ across the age at quit distribution of FS. We used data from Wave 1 (2008) of the National Income Dynamics Study to characterize the distribution of age at quit among FS in South Africa [6]. We considered respondents who reported previously smoking cigarettes regularly but no current use to be FS, considering the reported age when they last smoked cigarettes to be their age at quit. We calculated the average value of $f$ among FS of a given age using the following equation:

$$\begin{aligned} \bar{f}=\frac{\sum_{i} w_{i}f\left( q_{i} \right)}{\sum_{i} w_{i}}\#\left( 4 \right) \end{aligned}$$

where $w_{i}$ is the respondent’s calibrated sample weight, $q_{i}$ is the reported age when the respondent last smoked cigarettes, and $f\left( q_{i} \right)$ is the value of $f$ calculated based on the respondent’s age at quit and the age-at-quit-stratified values shown in Table S3. We used equation (4) to calculate $\bar{f}$ based on a final sample of 568 FS, which we stratified into 10-year age brackets. Calculated values of $\bar{f}$ are displayed in Table S7.

**Smoking prevalence by age, sex, and HIV status**

We estimated the prevalence of CS, FS, and NS in South Africa stratified by age, sex, and HIV status based on data from the 2016 South Africa Demographic and Health Survey (SADHS) [1]. We considered those who reported daily or occasional use of cigarettes to be CS and those who reported no history of tobacco use to be NS. Any respondents not categorized as CS or NS were assumed to be FS. We calculated the prevalence of CS, FS, and NS among the general population in South Africa stratified by age and sex using the male and female sample weights provided as part of the SADHS dataset. We also calculated smoking prevalence by age, sex, and HIV status using a separate set of sample weights specific to those selected for HIV testing (Table S4). Population averages were calculated using the Survey R package [7].

## **Derivation of non-AIDS mortality rates for CS, FS, and NS**

To derive non-AIDS mortality rates stratified by smoking status, we assumed that the overall non-AIDS mortality rate ($M_{all}$) is a weighted average of the non-AIDS mortality rates for NS, FS, and CS.

$$\begin{aligned} M_{all}=M_{N}P_{N}+\bar{M_{F}}P_{F}+M_{C}P_{C}.\#\left( 5 \right) \end{aligned}$$

We estimated the overall non-AIDS mortality rate among PWH in South Africa, stratified by age and sex, based on population estimates and cause-specific death data reported by the United Nations and the World Health Organization [8,9]. Next, we assumed that the fraction of non-AIDS deaths attributable to smoking is equal to the relative difference in non-AIDS mortality rates between NS with HIV and the overall population of PWH (the attributable fraction [$AF$]).

$$\begin{aligned} AF=\frac{M_{all}-M_{N}}{M_{all}}=\frac{M_{N}P_{N}+\bar{M_{F}}P_{F}+M_{C}P_{C}-M_{N}}{M_{N}P_{N}+\bar{M_{F}}P_{F}+M_{C}P_{C}}.\#\left( 6 \right) \end{aligned}$$

We assumed this attributable fraction was equal to estimates of the fraction of all-cause deaths in South Africa attributable to smoking reported by the Global Burden of Disease study, stratified by age and sex (Table S2) [10].

Finally, we assumed the average non-AIDS mortality rate among FS within a given age and sex category was a linear combination of the rates for NS and CS:

$$\begin{aligned} \bar{M_{F}}=M_{N}+\left( M_{C}-M_{N} \right)\bar{f},\#\left( 7 \right) \end{aligned}$$

where $\bar{f}$ is the average fraction of excess mortality risk retained by FS after accounting for the age at quit distribution of FS in South Africa, as described above (Table S3, Table S7) [5,6]. Equations (5)-(7) form a system of linear equations with three unknowns ($M_{N}$, $\bar{M_{F}}$, and $M_{C}$). This system can be written in the form $\boldsymbol{Ax=b}$, where $\boldsymbol{A}$ is the matrix of coefficients of the unknown variables, $\boldsymbol{x}$ is the vector of unknowns, and $\boldsymbol{b}$ is a vector of constants.

$$\begin{aligned} \underset{\boldsymbol{A}}{\underbrace{\left[ \begin{matrix} P_{N} & P_{F} & P_{C} \\ P_{N}\left( 1-AF \right)-1 & P_{F}\left( 1-AF \right) & P_{C}\left( 1-AF \right) \\ 1-\bar{f} & -1 & \bar{f} \end{matrix} \right]}}\underset{\boldsymbol{x}}{\underbrace{\left[ \begin{matrix} M_{N} \\ \bar{M_{F}} \\ M_{C} \end{matrix} \right]}}=\underset{\boldsymbol{b}}{\underbrace{\left[ \begin{matrix} M_{all} \\ 0 \\ 0 \end{matrix} \right]}}\#\left( 8 \right) \end{aligned}$$

To solve for our unknown variables, we can multiply both sides of equation (8) by the inverse of matrix $\boldsymbol{A}$:

$$\begin{aligned} \boldsymbol{x}\boldsymbol{=}\boldsymbol{A}^{-1}\boldsymbol{b}\boldsymbol{\#}\left( 9 \right) \end{aligned}$$

After solving, we may write the following equations for the non-AIDS mortality rate among NS, FS, and CS:

$$\begin{aligned} M_{N}=M_{all}\left( 1-AF \right)\#\left( 10 \right) \end{aligned}$$

$$\begin{aligned} \bar{M_{F}}=\frac{M_{all}\left( P_{C}\left( 1-AF \right)\left( 1-\bar{f} \right)+\left( 1-P_{N}\left( 1-AF \right) \right)\bar{f} \right)}{P_{C}+P_{F}\bar{f}}=M_{N}+\left( M_{C}-M_{N} \right)\bar{f}\#\left( 11 \right) \end{aligned}$$

$$\begin{aligned} M_{C}=\frac{M_{all}\left( 1-P_{F}\left( 1-AF \right)\left( 1-\bar{f} \right)-P_{N}\left( 1-AF \right) \right)}{P_{C}+P_{F}\bar{f}}\#\left( 12 \right) \end{aligned}$$

Equations (10) and (12) are used to determine mortality rates when performing simulations for NS and CS, respectively. When assessing the benefits of smoking cessation among specific population sub-groups, we determined mortality rates for FS based on their age at quit:

$$\begin{aligned} M_{F}=M_{N}+\left( M_{C}-M_{N} \right)f\left( q \right)\#\left( 13 \right) \end{aligned}$$

where $f\left( q \right)$ is the fraction of excess non-AIDS mortality risk retained by FS based on their age at quit (Table S3).

## **Model validation methods**

For internal validation, within each of 6 subgroups (3 initial ages [35/45/55y] and 2 sexes [females/males]) of PWH, we calculated life expectancy (LE) based on proportion-weighted LE of CS, FS, and NS using smoking prevalence data from SADHS [1]. We compared this weighted average LE with the LE generated from a CEPAC model simulation of PWH that did not stratify by smoking status – i.e., no distinction between CS, FS, and NS. For external validation, we compared base case cohort and ART-naïve cohort CEPAC LE projections (not stratified by smoking status) with the LE for: 1) PWH in South Africa with virologic suppression on ART from 2015 to 2018 [2], and 2) PWH in South Africa who started ART between 2001-2010 and survived the first two years on ART [3]. Results of the validation exercises are described in the Supplementary Results and Table S6.

**Supplementary Results**

## **Model validation results**

In external validation exercises, base case and ART naïve CEPAC LE projections lie between Payne et al. estimates as an upper bound, and Johnson et al. estimates as a lower bound (Table S6) [2,3]. Base case CEPAC LE projections for females and males aged 45y are 3.1 and 1.6 years less than Payne et al. LE projections. Though both CEPAC base case cohorts and Payne et al. focus on virologically suppressed PWH in South Africa, CEPAC cohorts can experience subsequent viremia and loss to follow-up from HIV care which thereby reduce LE. Base case and ART naïve CEPAC LE projections are greater than Johnson et al. LE projections by 1.9-4.0 life-years and 0.1-2.4 life-years, respectively. We would anticipate greater LE among CEPAC cohorts compared to Johnson et al. due to higher CD4 counts at presentation to care and access to more effective ART (TLD) compared to the 2001-2010 period.

# **Supplementary References**

1. National Department of Health (NDoH), Statistics South Africa (Stats SA), South African Medical Research Council (SAMRC), and ICF. South Africa Demographic and Health Survey, 2016 [Internet]. 2017. Available from: https://dhsprogram.com/methodology/survey/survey-display-390.cfm

2. Payne CF, Houle B, Chinogurei C, Herl CR, Kabudula CW, Kobayashi LC, et al. Differences in healthy longevity by HIV status and viral load among older South African adults: an observational cohort modelling study. Lancet HIV. 2022 Oct;9(10):e709–16.

3. Johnson LF, Mossong J, Dorrington RE, Schomaker M, Hoffmann CJ, Keiser O, et al. Life expectancies of South African adults starting antiretroviral treatment: collaborative analysis of cohort studies. PLoS Med. 2013 Apr 9;10(4):e1001418.

4. Yapa HM, Kim HY, Petoumenos K, Post FA, Jiamsakul A, De Neve JW, et al. CD4+ T-cell count at antiretroviral therapy initiation in the “treat-all” era in rural South Africa: an interrupted time series analysis. Clin Infect Dis. 2022 Apr 15;74(8):1350–9.

5. Jha P, Ramasundarahettige C, Landsman V, Rostron B, Thun M, Anderson RN, et al. 21st-century hazards of smoking and benefits of cessation in the United States. N Engl J Med. 2013 Jan 24;368(4):341–50.

6. Southern Africa Labour and Development Research Unit. National Income Dynamics Study 2008, Wave 1 [Internet]. DataFirst; 2008 [cited 2024 May 10]. Available from: https://www.datafirst.uct.ac.za/dataportal/index.php/catalog/451

7. Lumley T. Survey: analysis of complex survey samples [Internet]. 2021. Available from: https://cran.r-project.org/web/packages/survey/survey.pdf

8. World population prospects - Population division - United Nations [Internet]. 2016 [cited 2024 May 10]. Available from: https://population.un.org/wpp/Download/Standard/Population/

9. Mortality estimates by cause, age, and sex for WHO member states. [Internet]. 2016 [cited 2024 May 10]. Available from: https://www.who.int/data/gho/data/themes/mortality-and-global-health-estimates/ghe-leading-causes-of-death

10. Global burden of 87 risk factors in 204 countries and territories, 1990–2019: a systematic analysis for the Global Burden of Disease Study 2019. Lancet. 2020 Oct 17;396(10258):1223–49.

11. Paton NI, Kityo C, Hoppe A, Reid A, Kambugu A, Lugemwa A, et al. Assessment of second-line antiretroviral regimens for HIV therapy in Africa. N Engl J Med. 2014 Jul 17;371(3):234–47.

12. Aboud M, Kaplan R, Lombaard J, Zhang F, Hidalgo JA, Mamedova E, et al. Dolutegravir versus ritonavir-boosted lopinavir both with dual nucleoside reverse transcriptase inhibitor therapy in adults with HIV-1 infection in whom first-line therapy has failed (DAWNING): an open-label, non-inferiority, phase 3b trial. Lancet Infect Dis. 2019 Mar 1;19(3):253–64.

13. Cheng Y, Sauer B, Zhang Y, Nickman NA, Jamjian C, Stevens V, et al. Adherence and virologic outcomes among treatment-naïve veteran patients with human immunodeficiency virus type 1 infection. Medicine (Baltimore). 2018 Jan 12;97(2):e9430.

14. Hakim JG, Thompson J, Kityo C, Hoppe A, Kambugu A, Oosterhout JJ van, et al. Lopinavir plus nucleoside reverse-transcriptase inhibitors, lopinavir plus raltegravir, or lopinavir monotherapy for second-line treatment of HIV (EARNEST): 144-week follow-up results from a randomised controlled trial. Lancet Infect Dis. 2018 Jan 1;18(1):47–57.

15. Viswanathan S, Justice AC, Alexander GC, Brown TT, Gandhi NR, McNicholl IR, et al. Adherence and HIV RNA suppression in the current era of highly active antiretroviral therapy. J Acquir Immune Defic Syndr. 2015 Aug 1;69(4):493–8.

16. Gachara G, Mavhandu LG, Rogawski ET, Manhaeve C, Bessong PO. Evaluating adherence to antiretroviral therapy using pharmacy refill records in a rural treatment site in South Africa. AIDS Res Treat. 2017;2017:5456219.

17. Grinsztejn B, Hughes MD, Ritz J, Salata R, Mugyenyi P, Hogg E, et al. Third-line antiretroviral therapy in low and middle income countries: ACTG A5288, a prospective strategy study. Lancet HIV. 2019 Sep;6(9):e588–600.

18. Pepperrell T, Venter WDF, McCann K, Bosch B, Tibbatts M, Woods J, et al. Participants on dolutegravir resuppress human immunodeficiency virus RNA after virologic failure: updated data from the ADVANCE trial. Clin Infect Dis. 2021 Aug 16;73(4):e1008–10.

19. Thun MJ, Carter BD, Feskanich D, Freedman ND, Prentice R, Lopez AD, et al. 50-year trends in smoking-related mortality in the United States. N Engl J Med. 2013 Jan 24;368(4):351–64.

20. Venter WDF, Sokhela S, Simmons B, Moorhouse M, Fairlie L, Mashabane N, et al. Dolutegravir with emtricitabine and tenofovir alafenamide or tenofovir disoproxil fumarate versus efavirenz, emtricitabine, and tenofovir disoproxil fumarate for initial treatment of HIV-1 infection (ADVANCE): week 96 results from a randomised, phase 3, non-inferiority trial. Lancet HIV. 2020 Oct 1;7(10):e666–76.

21. NAMSAL ANRS 12313 Study Group. Dolutegravir-based or low-dose efavirenz–based regimen for the treatment of HIV-1. N Engl J Med. 2019 Aug 29;381(9):816–26.

22. Kaplan SR, Oosthuizen C, Stinson K, Little F, Euvrard J, Schomaker M, et al. Contemporary disengagement from antiretroviral therapy in Khayelitsha, South Africa: A cohort study. PLoS Med. 2017 Nov 7;14(11):e1002407.

23. Sitas F, Egger S, Bradshaw D, Groenewald P, Laubscher R, Kielkowski D, et al. Differences among the coloured, white, black, and other South African populations in smoking-attributed mortality at ages 35-74 years: a case-control study of 481,640 deaths. Lancet. 2013 Aug 24;382(9893):685–93.

24. Wong EB, Olivier S, Gunda R, Koole O, Surujdeen A, Gareta D, et al. Convergence of infectious and non-communicable disease epidemics in rural South Africa: a cross-sectional, population-based multimorbidity study. Lancet Glob Health. 2021 Jul;9(7):e967–76.

# **Supplementary Tables**

## **Table S1. Additional HIV-related input parameters.**

| **Parameter** | **Value** | | **Source** |  |
| --- | --- | --- | --- | --- |
|  | **Base case** | **ART-naïve** |  |  |
| **Efficacy of 2^nd^ -line ART (AZT + 3TC + LPV/r)** |  |  |  |  |
| Average HIV RNA suppression at 48 weeks, % | 72.3 | 68.6 |  |  |
| Adherence > 87% | 75.4% | -- | [11,12] |  |
| Adherence 68%-87% | 70.5% | -- | [13] |  |
| Adherence < 68% | 0% | -- | Assumption |  |
| Average monthly probability of subsequent viremia | 0.2 | 0.2 |  |  |
| Adherence > 95% | 0.2% | -- | [11,14] |  |
| Adherence 30%-95% | 0.3% | -- | [15] |  |
| Adherence < 30% | 18.0% | -- | [16] |  |
| **Efficacy of 3^rd^ -line ART (DRV/r + DTG + 3TC + ABC)** |  |  |  |  |
| Average HIV RNA suppression at 48 weeks, % | 85.9 | 80.9 |  |  |
| Adherence > 95% | 90.0% | -- | [17] |  |
| Adherence 71%-95% | 83.9% | -- | [13] |  |
| Adherence < 71% | 0% | -- | Assumption |  |
| Average monthly probability of subsequent viremia | 0.4 | 0.4 |  |  |
| Adherence > 95% | 0.4% | -- | [17] |  |
| Adherence 30%-95% | 0.5% | -- | [15] |  |
| Adherence < 30% | 18.0% | -- | [16] |  |
| **Proportion of patients who attain virologic suppression on ART after resuppression attempt** | 67.0% | -- | [18] |  |
| Abbreviations: ART, antiretroviral therapy; AZT, zidovudine; 3TC, lamivudine; LPV/r, lopinavir/ritonavir; DRV/r, darunavir/ritonavir; DTG, dolutegravir; ABC, abacavir. | | | |  |
|  | | | |  |

## **Table S2. Fraction of deaths attributable to smoking and non-AIDS mortality hazard ratios for people with current vs. never smoking status in South Africa.**

| **Age group, years** | **Fraction of deaths attributable to smoking as reported by the Global Burden of Disease study**[10] | | **Calculated non-AIDS mortality hazard ratios for people with current vs. never smoking status^a^** | |
| --- | --- | --- | --- | --- |
|  | **Females** | **Males** | **Females** | **Males** |
| 40-44 | 0.016 | 0.051 | 1.2 | 1.1 |
| 45-49 | 0.029 | 0.073 | 1.4 | 1.2 |
| 50-54 | 0.050 | 0.120 | 1.4 | 1.3 |
| 55-59 | 0.071 | 0.165 | 1.6 | 1.4 |
| 60-64 | 0.098 | 0.202 | 2.0 | 1.7 |
| 65-69 | 0.098 | 0.197 | 2.0 | 1.7 |
| 70-74 | 0.102 | 0.191 | 2.3 | 1.9 |
| 75-79 | 0.077 | 0.158 | 2.0 | 1.7 |
| 80-84 | 0.075 | 0.137 | 1.9 | 1.6 |
| 85-89 | 0.054 | 0.107 | 1.6 | 1.5 |
| 90-94 | 0.046 | 0.089 | 1.6 | 1.4 |
| 95-99 | 0.040 | 0.078 | 1.5 | 1.3 |

^a^The fraction of deaths attributable to smoking were used in the derivation of smoking-stratified non-AIDS mortality rates. To derive mortality hazard ratios, we compared the resulting mortality rates for people with current vs. never smoking status.

## **Table S3. Smoking-associated non-AIDS mortality hazard ratios and fractional risk retained after smoking cessation, based on age at cessation.**

| **Smoking status** | **Adjusted all-cause mortality hazard ratios vs NS reported by Jha et al.**[5] | **Fraction of excess smoking-associated non-AIDS mortality risk retained by FS after quitting (**$\boldsymbol{f}$**)^a^** |
| --- | --- | --- |
| Never | Reference | -- |
| Former |  |  |
| Quit at ages <25 years | 1.0 | 0.00 |
| Quit at ages 25-34 years | 1.0 | 0.00 |
| Quit at ages 35-44 years | 1.2 | 0.11^a^ |
| Quit at ages 45-54 years | 1.5 | 0.26 |
| Quit at ages 55-64 years | 1.7 | 0.37 |
| Quit at ages >64 years | Not reported | 0.37^b^ |
| Current (continue to smoke) | 2.9 | -- |

Abbreviations: NS, people with never smoking status; FS, people with former smoking status.

^a^We assumed the fraction of deaths attributable to smoking is 0% among those aged less than 40 years. Additionally, the fraction of excess non-AIDS mortality risk retained by FS was set to zero among people who quit prior to 40 years of age. Both of these are in accordance with previously described data and methods [5,19].

^b^Jha et al. did not report an all-cause mortality hazard ratio for FS who quit at ages >64 years [5]. We assumed that the fraction of excess non-AIDS mortality risk retained by this group would be the same as in those who quit at ages 55-64 years.

Table S4. Smoking prevalence in South Africa based on data from the 2016 South Africa Demographic and Health Survey [1].

| **Population/ Sex / Age** | **Smoking status prevalence, %** | | |
| --- | --- | --- | --- |
|  | **Current** | **Former** | **Never** |
| **General population^a^** |  |  |  |
| Females |  |  |  |
| 15-19 years | 4.1 | 1.2 | 94.7 |
| 20-29 years | 6.3 | 1.7 | 92.0 |
| 30-39 years | 8.0 | 1.2 | 90.8 |
| 40-49 years | 7.9 | 1.2 | 90.9 |
| 50-59 years | 12.0 | 2.2 | 85.8 |
| 60-69 years | 10.2 | 3.1 | 86.7 |
| ≥70 years | 8.1 | 3.7 | 88.2 |
| Males |  |  |  |
| 15-19 years | 18.2 | 4.3 | 77.5 |
| 20-29 years | 42.7 | 4.5 | 52.8 |
| 30-39 years | 40.9 | 6.5 | 52.6 |
| 40-49 years | 45.4 | 9.2 | 45.4 |
| 50-59 years | 44.5 | 7.5 | 48.0 |
| 60-69 years | 33.3 | 19.8 | 46.9 |
| ≥70 years | 22.7 | 15.3 | 62.0 |
| **People with HIV** |  |  |  |
| Females |  |  |  |
| 15-29 years | 5.5 | 0.7 | 93.8 |
| 30-39 years | 9.0 | 1.6 | 89.4 |
| 40-49 years | 4.5 | 0.7 | 94.8 |
| ≥50 years | 8.7 | 1.0 | 90.3 |
| Males |  |  |  |
| 15-29 years | 43.6 | 2.6 | 53.8 |
| 30-39 years | 51.4 | 5.9 | 42.8 |
| 40-49 years | 46.1 | 5.8 | 48.1 |
| ≥50 years | 31.8 | 7.4 | 60.8 |
| **People without HIV** |  |  |  |
| Females |  |  |  |
| 15-29 years | 4.2 | 1.5 | 94.4 |
| 30-39 years | 10.7 | 0.9 | 88.4 |
| 40-49 years | 11.4 | 1.1 | 87.5 |
| ≥50 years | 12.1 | 3.0 | 84.8 |
| Males |  |  |  |
| 15-29 years | 33.3 | 4.5 | 62.2 |
| 30-39 years | 34.2 | 7.2 | 58.6 |
| 40-49 years | 41.4 | 14.2 | 44.4 |
| ≥50 years | 35.2 | 15.9 | 48.9 |

^a^Age brackets were chosen to ensure a minimum of 50 respondents per age group.

Table S5. One-way sensitivity and scenario analysis results: model-projected life expectancy when varying smoking and HIV-related parameters.

| Age at model start and smoking status | Base case^a^ | | FS quit smoking after 2y | | FS quit smoking after 5y | | FS quit smoking after 10y | | Sitas et al. smoking-associated mortality HRs^b^ | | Jha et al. smoking-associated mortality HRs^c^ | | Smoking prevalence estimates for PWH from SADHS^d^ | | Complete ART adherence | | Initial CD4 cell count 100/µL | | Initial CD4 cell count 400/µL | | Initial CD4 cell count 800/µL | | Higher disengage-ment from care | | ART-naive | |
| --- | --- | --- | --- | --- | --- | --- | --- | --- | --- | --- | --- | --- | --- | --- | --- | --- | --- | --- | --- | --- | --- | --- | --- | --- | --- | --- |
|  | F | M | F | M | F | M | F | M | F | M | F | M | F | M | F | M | F | M | F | M | F | M | F | M | F | M |
| **35 years** |  |  |  |  |  |  |  |  |  |  |  |  |  |  |  |  |  |  |  |  |  |  |  |  |  |  |
| Current | 65.8 | 62.0 | -^e^ | - | - | - | - | - | 68.6 | 63.0 | 62.5 | 60.1 | 65.2 | 61.8 | 68.7 | 64.6 | 57.0 | 55.6 | 64.0 | 61.5 | 66.6 | 63.6 | 62.5 | 59.0 | 63.8 | 60.6 |
| Former | 70.5 | 65.2 | 70.5 | 65.2 | 69.7 | 64.7 | 68.8 | 64.0 | 70.1 | 64.4 | 71.3 | 67.8 | 70.5 | 65.2 | 74.8 | 68.7 | 59.8 | 57.7 | 68.1 | 64.6 | 71.5 | 67.2 | 65.9 | 61.3 | 67.8 | 63.4 |
| Never | 70.5 | 65.2 | - | - | - | - | - | - | 70.1 | 64.4 | 71.3 | 67.8 | 70.5 | 65.2 | 74.8 | 68.7 | 59.8 | 57.7 | 68.1 | 64.6 | 71.5 | 67.2 | 65.9 | 61.3 | 67.8 | 63.5 |
| *Life-years gained from smoking cessation* | 4.7 | 3.2 | 4.7 | 3.2 | 4.0 | 2.7 | 3.0 | 2.0 | 1.5 | 1.5 | 8.8 | 7.8 | 5.4 | 3.4 | 6.1 | 4.1 | 2.8 | 2.1 | 4.2 | 3.1 | 4.9 | 3.6 | 3.3 | 2.3 | 4.0 | 2.8 |
| **45 years** |  |  |  |  |  |  |  |  |  |  |  |  |  |  |  |  |  |  |  |  |  |  |  |  |  |  |
| Current | 68.9 | 65.3 | - | - | - | - | - | - | 72.1 | 66.5 | 65.6 | 63.4 | 68.2 | 65.2 | 70.5 | 66.7 | 62.6 | 61.0 | 67.7 | 65.1 | 69.4 | 66.3 | 67.0 | 63.7 | 67.8 | 64.7 |
| Former | 72.3 | 67.7 | 72.2 | 67.6 | 72.1 | 67.5 | 71.2 | 66.8 | 73.1 | 67.5 | 70.7 | 67.8 | 72.0 | 67.7 | 74.7 | 69.6 | 64.8 | 62.6 | 70.8 | 67.4 | 73.0 | 68.9 | 69.7 | 65.5 | 70.8 | 66.8 |
| Never | 74.2 | 69.0 | - | - | - | - | - | - | 73.7 | 68.1 | 75.1 | 71.8 | 74.2 | 69.0 | 77.0 | 71.2 | 66.0 | 63.5 | 72.5 | 68.7 | 75.0 | 70.4 | 71.2 | 66.6 | 72.5 | 68.1 |
| *Life-years gained from smoking cessation* | 3.4 | 2.4 | 3.3 | 2.3 | 3.2 | 2.2 | 2.3 | 1.5 | 1.1 | 1.0 | 5.1 | 4.4 | 3.7 | 2.5 | 4.1 | 2.9 | 2.1 | 1.6 | 3.1 | 2.3 | 3.6 | 2.6 | 2.7 | 1.9 | 3.0 | 2.1 |
| **55 years** |  |  |  |  |  |  |  |  |  |  |  |  |  |  |  |  |  |  |  |  |  |  |  |  |  |  |
| Current | 72.1 | 69.0 | - | - | - | - | - | - | 75.7 | 70.5 | 69.4 | 67.3 | 71.6 | 68.9 | 72.9 | 69.7 | 68.2 | 66.5 | 71.5 | 68.9 | 72.4 | 69.5 | 71.2 | 68.2 | 71.9 | 69.2 |
| Former | 74.9 | 70.9 | 74.6 | 70.6 | 74.3 | 70.4 | 73.6 | 69.9 | 76.5 | 71.2 | 72.9 | 70.2 | 74.5 | 70.8 | 76.1 | 71.8 | 70 | 67.8 | 74 | 70.8 | 75.3 | 71.6 | 73.5 | 69.8 | 74.2 | 70.8 |
| Never | 77.9 | 73.1 | - | - | - | - | - | - | 77.3 | 72 | 78.7 | 75.4 | 77.9 | 73.1 | 79.5 | 74.3 | 71.9 | 69.4 | 76.8 | 72.9 | 78.4 | 74 | 76.1 | 71.7 | 77 | 72.9 |
| *Life-years gained from smoking cessation* | 2.8 | 1.9 | 2.5 | 1.7 | 2.2 | 1.4 | 1.5 | 0.9 | 0.8 | 0.7 | 3.5 | 2.9 | 2.9 | 1.9 | 3.2 | 2.2 | 1.8 | 1.3 | 2.6 | 1.9 | 2.9 | 2.1 | 2.4 | 1.6 | 2.4 | 1.6 |

Abbreviations: FS, people with former smoking status; HRs, hazard ratios; PWH, people with HIV; SADHS, South Africa Demographic and Health Survey; F, females; M, males.

^a^ People with HIV enter the model with virologic suppression, though there is a probability of subsequent virologic rebound. Mean adherence is 95.3%, and there is a 0.7% monthly probability of disengagement from HIV care [20–22]. FS quit at model start.

^b^In this simulation we used a non-AIDS mortality HR of 1.2 for current vs. never smoking, as reported by Sitas et al [23].

^c^In this simulation we used a non-AIDS mortality HR of 3.0 (females) and 2.8 (males) for current vs. never smoking, as reported by Jha et al [5].

^d^In this simulation we used SADHS smoking prevalence estimates specific to PWH for calculations of smoking-stratified non-AIDS mortality. In base case simulations, we used SADHS smoking prevalence estimates for the general population because the larger sample size allowed for greater precision when stratifying by age and sex [1].

^e^Cells containing “-“ do not differ from the base case.

## **Table S6. Model validation exercises: CEPAC life expectancy projections and life expectancy estimates from other studies in South Africa.**

| **Sex/Age** | **Payne et al. (2022): virologically suppressed PWH**[2] | **Johnson et al. (2013): PWH who initiate ART at the stated age and survive at least two years**[3] | **CEPAC projections: PWH virologically suppressed at model start^a^** | | **CEPAC projections: PWH who initiate ART at the stated age (ART-naïve) and survive at least two years^b^** | |
| --- | --- | --- | --- | --- | --- | --- |
|  |  |  | **Weighted Average^c^** | **All PWH^d^** | **Weighted Average^c^** | **All PWH^d^** |
| **Females** |  |  |  |  |  |  |
| 35 years | -^e^ | 67.4 | 70.1 | 69.9 | 67.5 | 67.3 |
| 45 years | 76.6 | 70.9 | 73.8 | 73.5 | 72.1 | 71.9 |
| 55 years | - | 74.6 | 77.1 | 77.1 | 76.4 | 76.4 |
| **Males** |  |  |  |  |  |  |
| 35 years | - | 59.9 | 63.9 | 63.9 | 62.3 | 62.3 |
| 45 years | 69.1 | 64.5 | 67.2 | 67.5 | 66.4 | 66.7 |
| 55 years | - | 69.5 | 71.1 | 71.4 | 71.1 | 71.4 |
|  |  |  |  |  |  |  |
| Abbreviations: LE, life expectancy; PWH, people with HIV; ART, antiretroviral therapy.  ^a^PWH enter the model with virologic suppression, though there is a probability of subsequent viremia and loss to follow-up from HIV care. Mean CD4 (standard deviation) at model start is 789 (318) cells/µl for females and 600 (303) cells/µl for males [24].  ^b^PWH initiate ART at model start. Mean CD4 (standard deviation) at model start is 432 (206) for females and 314 (160) for males [4]. ^c^LE was calculated using the prevalence-weighted average of life expectancies of separate simulated cohorts of people with current smoking status, former smoking status, and never smoking status. ^d^LE for a simulated cohort of all PWH, not stratified by smoking status.  ^e^Payne et al. report LE only for those aged 45y | | | | | | |

Table S7. Characteristics of people with former smoking status in South Africa based on data from the National Income Dynamics Study [6].

| **Age group** | **Average^a^ current age, years** | **Average age at time of quitting smoking, years** | **Average fraction of excess non-AIDS mortality risk retained by FS at the population level in South Africa (**$\bar{\boldsymbol{f}}$**)^b^** |
| --- | --- | --- | --- |
| 20-29 years  (n=89) | 24.7 | 21.5 | 0.00 |
| 30-39 years  (n=111) | 34.9 | 26.3 | 0.00 |
| 40-49 years  (n=109) | 44.4 | 34.1 | 0.04 |
| 50-59 years  (n=107) | 54.0 | 39.1 | 0.11 |
| ≥60 years  (n=152) | 66.8 | 44.4 | 0.18 |
| Abbreviations: FS, people with former smoking status.  ^a^Population averages were calculated using the calibrated sample weights included as part of the National Income Dynamics Study (NIDS) dataset.  ^b^The average fraction of excess non-AIDS mortality risk retained by FS at the population level in South Africa ($\bar{f}$) was calculated based on the age-at-quit-stratified values of $f$ displayed in Table S3 and the distribution of age at quit in the National Income Dynamics Study dataset according to equation (4) in the Supplementary Methods. | | | |
